# Diet-induced obesity differentially regulates behavioral, biomechanical, and molecular risk factors for osteoarthritis in mice

**DOI:** 10.1186/ar3068

**Published:** 2010-07-06

**Authors:** Timothy M Griffin, Beverley Fermor, Janet L Huebner, Virginia B Kraus, Ramona M Rodriguiz, William C Wetsel, Li Cao, Lori A Setton, Farshid Guilak

**Affiliations:** 1 Department of Surgery, Duke University Medical Center, 375 Medical Sciences Research Building, Durham, NC 27710, USA; 2 Department of Medicine, Duke University Medical Center, 2100 Erwin Road, Durham, NC 27710, USA; 3 Department of Psychiatry and Behavioral Sciences, Mouse Behavioral and Neuroendocrine Analysis Core Facility, Duke University Medical Center, 2100 Erwin Road, Durham, NC 27710, USA; 4 Departments of Neurobiology and Cell Biology, Duke University Medical Center, 2100 Erwin Road, Durham, NC 27710, USA; 5 Department of Biomedical Engineering, Duke University, 136 Hudson Hall, Durham, NC 27708, USA; 6 Current address: Program in Free Radical Biology and Aging, Oklahoma Medical Research Foundation, Biochemistry and Molecular Biology, University of Oklahoma Health Science Center, 825 NE 13th St, Oklahoma City, OK 73104 USA

## Abstract

**Introduction:**

Obesity is a major risk factor for the development of osteoarthritis in both weight-bearing and nonweight-bearing joints. The mechanisms by which obesity influences the structural or symptomatic features of osteoarthritis are not well understood, but may include systemic inflammation associated with increased adiposity. In this study, we examined biomechanical, neurobehavioral, inflammatory, and osteoarthritic changes in C57BL/6J mice fed a high-fat diet.

**Methods:**

Female C57BL/6J mice were fed either a 10% kcal fat or a 45% kcal fat diet from 9 to 54 weeks of age. Longitudinal changes in musculoskeletal function and inflammation were compared with endpoint neurobehavioral and osteoarthritic disease states. Bivariate and multivariate analyses were conducted to determine independent associations with diet, percentage body fat, and knee osteoarthritis severity. We also examined healthy porcine cartilage explants treated with physiologic doses of leptin, alone or in combination with IL-1α and palmitic and oleic fatty acids, to determine the effects of leptin on cartilage extracellular matrix homeostasis.

**Results:**

High susceptibility to dietary obesity was associated with increased osteoarthritic changes in the knee and impaired musculoskeletal force generation and motor function compared with controls. A high-fat diet also induced symptomatic characteristics of osteoarthritis, including hyperalgesia and anxiety-like behaviors. Controlling for the effects of diet and percentage body fat with a multivariate model revealed a significant association between knee osteoarthritis severity and serum levels of leptin, adiponectin, and IL-1α. Physiologic doses of leptin, in the presence or absence of IL-1α and fatty acids, did not substantially alter extracellular matrix homeostasis in healthy cartilage explants.

**Conclusions:**

These results indicate that diet-induced obesity increases the risk of symptomatic features of osteoarthritis through changes in musculoskeletal function and pain-related behaviors. Furthermore, the independent association of systemic adipokine levels with knee osteoarthritis severity supports a role for adipose-associated inflammation in the molecular pathogenesis of obesity-induced osteoarthritis. Physiologic levels of leptin do not alter extracellular matrix homeostasis in healthy cartilage, suggesting that leptin may be a secondary mediator of osteoarthritis pathogenesis.

## Introduction

Osteoarthritis is a progressive, age-related disease characterized by cartilage destruction and abnormal bone remodeling, resulting in joint pain and severe disability. The etiology of this disease is complex and multifaceted, and numerous genetic and environment risk factors have been identified that modify disease incidence and severity. One of the most significant risk factors is obesity. The association between obesity and osteoarthritis has been extensively studied; however, there is currently no comprehensive explanation for why obesity increases the risk of osteoarthritis at different sites throughout the body. At the knee joint, where obesity increases the risk of developing osteoarthritis by twofold to 10-fold [1,2], local biomechanical factors associated with body mass index, limb alignment, and quadriceps muscle strength can all influence both the onset and progression of knee osteoarthritis [3-5]. Nevertheless, these factors do not explain the association between obesity and osteoarthritis at nonload-bearing joints [2,6,7], and suggest that, in certain cases, systemic factors may be involved in the onset or progression of the disease.

Attempts to identify systemic versus local factors linking obesity and osteoarthritis, independent of weight-bearing biomechanical factors associated with body mass index, have generally been unsuccessful (for example, serum cholesterol, glucose, lipids, uric acid, blood pressure, or body fat distribution) [8-13]. Hart and colleagues were, however, able to show that hypertension, hypercholesterolemia, and increased blood glucose were associated with unilateral and bilateral knee osteoarthritis independent of obesity [14]. Obesity is associated with mild, chronic inflammation [15], suggesting that inflammatory molecules secreted from adipose tissue may provide a critical, nonbiomechanical link between obesity and osteoarthritis. Numerous proinflammatory cytokines that are secreted from hypertrophic abdominal adipose tissue (that is, adipokines or cytokines such as leptin, TNFα, IL-1, and IL-6) are elevated in osteoarthritic joints and can induce catabolic processes in chondrocytes *in vitro*, leading to extracellular matrix degradation. In particular, leptin has engendered intense interest because it upregulates both catabolic and anabolic activities of chondrocytes [16-18], consistent with cellular changes associated with osteoarthritis. In addition to effects of adipokines on chondrocyte matrix metabolism, adipokines and associated metabolic abnormalities may contribute to joint degeneration through impaired neuromuscular function that alters the mechanical environment of the joint. An integrative approach that encompasses changes in biomechanical and inflammatory factors associated with obesity thus represents a critical step in identifying the etiopathology of obesity-associated joint degeneration.

A primary clinical outcome of osteoarthritis is functional disability caused by chronic joint pain. There has been limited success, however, in predicting joint pain from pathological joint changes [19,20]. This limitation may be attributed to pain perception itself since it involves nociceptive factors that mediate the intensity of the afferent signal and cognitive factors that excite or suppress this nociceptive response [21,22]. Obesity in older adults is associated with increased prevalence and incidence of pain [23]; and in these patients with knee osteoarthritis, cognitive factors reduce the self-efficacy in pain management [24]. The relationship between reduced self-efficacy, which may occur with disorders of anxiety or depression, and psychological aspects of nociception associated with obesity is poorly understood and represents an opportunity to investigate behavioral and molecular risk factors relating joint structural changes to pain.

In the present study, we used a dietary model of obesity to address the integrated role of biomechanical and inflammatory factors in the pathogenesis of osteoarthritis, and we investigated the effect of dietary obesity on factors affecting pain-related behaviors in mice. When fed a high-fat diet, C57BL/6J mice develop changes associated with metabolic syndrome in humans including hyperglycemia, hyperinsulinemia, hypertension, and central adiposity [25]. It has been reported previously that C57BL mice develop early-onset osteoarthritis when fed a high-fat diet [26]. Little is known, however, about the mechanism by which dietary fat induces osteoarthritis or whether this strain of mice accurately models the pathogenesis of the human disease [27]. C57BL/6 mice vary in their susceptibility to diet-induced obesity [28]. We therefore exploited this variable dietary response to investigate the effect of a high-fat diet, with or without high adiposity, on characteristics of osteoarthritis. Based upon these findings, we examined independent and synergistic effects of adipokines and fatty acids on cartilage matrix homeostasis in a porcine cartilage explant model. We show that diet-induced obesity mediates the development of osteoarthritis in proportion to increases in adiposity and serum leptin concentration. We also demonstrate that a high-fat diet decreases motor performance and strength, causes thermal hyperalgesia, and alters coping-related behaviors in mice, indicating important dietary effects on motor function and pain responses. These findings are consistent with clinical studies of osteoarthritis and support the use of diet-induced obese mouse models to study behavioral and structural changes associated with osteoarthritis.

## Materials and methods

### Animals

All animal care and experimental procedures were conducted under an approved protocol from the Duke University Institutional Animal Care and Use Committee. Female C57BL/6J mice were purchased from The Jackson Laboratory (Bar Harbor, ME, USA). Mice were group-housed in filter-top cages with *ad libitum *access to water and chow. Mice were placed on either a high-fat diet (D12451, 45% kcal fat; Research Diets, New Brunswick, NJ, USA) or a control diet (D12450B, 10% kcal fat; Research Diets), beginning at 9 weeks of age. Animal weights were recorded weekly, and mice remained on their respective diets until the completion of the study at 54 weeks of age.

### Evaluation of osteoarthritis

Degenerative joint changes were evaluated by histological analysis and biomechanical measurements of cartilage compressive material properties. For histological analysis, intact knee joints were decalcified, dehydrated, and embedded in paraffin. Serial sagittal 6 μm sections were collected throughout the medial and lateral condyles. Sections were stained with hematoxylin, fast green, and safranin-O, and sections in the tibiofemoral cartilage-cartilage contact region from the medial and lateral condyles were scored for degenerative changes using a modified Mankin scoring system [29]. Briefly, this scoring system included changes in articular cartilage structure (0 to 11), safranin-O staining (0 to 8), tidemark duplication (0 to 3), fibrocartilage (0 to 2), chondrocyte clones in uncalcified cartilage (0 to 2), hypertrophic chondrocytes (0 to 2), and relative subchondral bone thickness (0 to 2) for a maximum score of 30 per location. Scores were determined by averaging values from three experienced, blinded graders for the summation of four locations in the joint: lateral femur, lateral tibia, medial femur, and medial tibia. Degenerative changes in the mandibular condyle of the temporomandibular joint were evaluated following this scoring and grading system, except that grading was restricted to changes in cartilage structure and safranin-O staining intensity.

Compressive cartilage material properties were determined by conducting a micro-indentation test of the medial tibial plateau using an electromechanical test system (ELF 3200; EnduraTEC, Minnetonka, MN, USA) instrumented with a low-capacity load cell (250 g; Sensotec, Columbus, OH, USA) and an extensometer (1 mm; Epsilon, Jackson, WY, USA) as described previously [30]. After applying a tare load of 0.15 g force and allowing it to equilibrate, a 0.2 g step load (ramping speed of 500 g/second) was applied to the cartilage surface and allowed to equilibrate for 200 seconds. Time, reaction force, and displacement data were collected at 1 Hz throughout the test. After mechanical testing, cartilage thickness was measured from the tissue surface to the calcified cartilage at a site adjacent to the test site, using previously described histological procedures. Indentation test results, together with a nonlinear optimization program employing a genetic algorithm for parameter estimation, were input into a biphasic finite element model of the micro-indentation test, which was used to obtain the biphasic, compressive material properties of tibial articular cartilage [30].

To quantify the effects of a high-fat diet on knee joint skeletal morphology, formalin-fixed joints were scanned using a microCT system (microCT 40; Scanco Medical AG, Basserdorf, Switzerland). A global thresholding procedure was used to segment calcified tissue from soft tissue. Linear attenuation values for the calcified tissue were scaled to bone density values (mg hydroxyapatite/cm^3^) using a hydroxyapatite calibration phantom. A direct three-dimensional approach in the epiphyseal region distal to the subchondral bone and proximal to the growth plate was applied to evaluate changes in the relative trabecular bone volume.

### Musculoskeletal function, gait, and spontaneous activity testing

Fore limb and hind limb grip strength were measured with a mouse grip strength meter (Ugo Basile, Varese, Italy) [31]. Grip strengths were measured after 13, 17, and 35 weeks of high-fat feeding. Motor learning, coordination, and endurance were assessed using a rotarod (Med-Associates, St Albans, VT, USA) with accelerating speed (4 to 40 rpm over 5 minutes) and constant speed (24 rpm) protocols [31]. Rotarod tests were conducted after 21 and 34 weeks of high-fat feeding.

Gait analysis was conducted during steady-speed spontaneous locomotion in a custom-built arena (25 cm × 75 cm) that contained a plexiglass bottom and a mirror positioned at 45° to allow simultaneous sagittal and ventral plane views. Spontaneous animal locomotion was recorded in the arena using a Motion Scope high-speed video camera (200 Hz; Red Lake Imaging Co., Tallahassee, FL, USA), and freely chosen speeds and stride frequencies were determined for each animal from three steady-speed locomotor bouts through the central 10 cm segment of the area. Gait tests were conducted after 10, 15, 21, 28, and 35 weeks of high-fat feeding. Gait kinetics were recorded in a custom arena fitted with a small force platform (AMTI, Watertown, MA, USA) that is capable of measuring the peak vertical ground reaction force in mice [32]. Hind limb vertical ground reaction forces and sagittal-plane high-speed video were recorded during spontaneous steady-speed locomotor bouts through the central 10 cm segment of the arena. Force-platform data were recorded after 41 weeks of high-fat feeding. Spontaneous locomotor activities in the open field (21 cm × 21 cm × 30 cm) were monitored by photobeams for 72 hours in an automated Omnitech Digiscan apparatus (AccuScan Instruments, Columbus, OH, USA) [31]. Light-phase and dark-phase locomotor activity (horizontal distance) was analyzed with the VersaMax program (AccuScan Instruments). Spontaneous locomotor activity was measured after 5, 11, 18, and 30 weeks of high-fat feeding.

### Cytokine and adipokine measurements

Blood was collected in anesthetized mice and dispensed into BD Vacutainer SST serum tubes (VWR, West Chester, PA, USA). After 30 minutes, tubes were centrifuged for 15 minutes at 3,500 rpm, and the serum was aliquoted for immediate storage at -80°C until analysis.

Levels of serum leptin were quantified by a sandwich ELISA specific for the mouse (Linco #EZML-82K; Billerica, MA, USA). Intra-assay and inter-assay coefficients of variation were 3% and 2.7%, respectively. Serum adiponectin concentrations were quantified by a sandwich ELISA specific for the mouse (Linco #EZMADP-60K; Billerica, MA, USA). Intra-assay and inter-assay coefficients of variation were 5.7% and 5.6%, respectively. IL-1α and IL-1-receptor antagonist serum levels were quantified by a quantitative sandwich ELISA developed specifically for the mouse (Quantikine #MLA00 and MRA00; R&D Systems, Minneapolis, MN, USA). Intra-assay and inter-assay coefficients of variation for IL-1α were 4.2% and 4.5%, respectively, and for IL-1-receptor antagonist were 2.4% and 5.7%, respectively.

The following cytokines and chemokines were measured in the serum using a 20-plex multiplex bead immunoassay (#LMC0006; Biosource, Carlsbad, CA, USA), specific to the mouse, with the Luminex 100 instrument: IL-1α, IL-1β, IL-2, IL-4, IL-6, IL-10, IL-12, IL-17, keratinocyte-derived cytokine (mouse analog of IL-8), IFNγ-induced protein, macrophage inflammatory protein-1α, and TNFα. All samples were analyzed as recommended by the manufacturer.

### Tissue culture experiments

Full-thickness articular cartilage explants were harvested from the femoral condyles of skeletally mature female pigs and were allowed to stabilize in culture for 72 hours at 37°C under 5% CO_2 _as previously described [33]. Explants were cultured in a 48-well plate containing 1 ml/well culture medium consisting of Dulbecco's low glucose modified Eagle medium (#11885-084; Invitrogen, Carlsbad, CA, USA) with 10% heat-inactivated FBS (Invitrogen), 0.1 mM nonessential amino acids (Invitrogen), 10 mM HEPES (Invitrogen), and 37.5 μg/ml ascorbate-2-phosphate (Sigma, St Louis, MO, USA). Explants were treated independently or in combination with recombinant human leptin (1, 10, and 100 ng/ml; Bachem, Torrance, CA, USA), porcine IL-1α (0.1 ng/ml; R&D Systems), or 1:1 palmitic:oleic fatty acids and l-carnitine (0.5 mM and 1 mM, respectively; Sigma-Aldrich, St. Louis, MO, USA) for 48 hours. Fatty acids were solublized in 1% BSA (fraction V; Sigma-Aldrich) and Dulbecco's low glucose modified Eagle medium. Then 1% BSA and 1 mM l-carnitine were added to control culture medium for fatty acid treatment experiments. Proteoglycan and protein synthesis rates were quantified simultaneously with leptin treatments by measuring the incorporation rates of [^35^S]sulfate (5 μCi/ml) and [^3^H]proline (20 μCi/ml), respectively. Explants were washed to remove unincorporated label and fully digested as previously described [33] prior to measuring disintegration rates. Total sulfated glycosaminoglycan (S-GAG) release into the media was measured with the 1,9-dimethylmethylene blue optical absorbance assay [33]. Nitric oxide production was quantified by measuring the amount of nitrite and nitrate (NO_x_) in the media using previously described methods and reagents [34].

### Affective behavioral trait measurements

Anxiety-like and depressive-like behaviors were evaluated in the animals following 40 weeks of high-fat feeding. The elevated zero maze was used to assess anxiety-like behaviors [31]. The maze consisted of a 5.5 cm wide circular (34 cm in diameter) black platform elevated 43 cm from the floor and was illuminated at ~60 lux. The maze comprised two open quadrants and two closed quadrants, all equal in size. The two closed quadrants were opposite each other and were enclosed by black walls 11 cm high. Mice were placed into a closed area and behaviors were videotaped for 5 minutes from a camera suspended 200 cm over the center of the maze.

Behavior was scored subsequently by trained observers, blind to the group assignment, using standard software (version 5.0; Noldus Information Technology, Leesburg, VA, USA). The behaviors included percentage time spent in the open areas, total numbers of transitions between the two open areas, stretch-attend postures, head-dipping behavior, and percentage time spent in freezing behavior. Depressive-like behaviors were examined by tail suspension [31]. Testing was conducted in a Med-Associates mouse tail suspension apparatus and analyzed using Threshold software. The day before testing, mice were tail marked and body weights were entered into the software program. For testing, mice were suspended by their tails for 6 minutes and time spent immobile was recorded.

### Thermal hyperalgesia experiments

Thermal sensitivity was evaluated using a sequential hotplate and tail flick test. For the hotplate test, an animal was placed on a hotplate (52 ± 1°C; Columbus Instruments, Columbus, OH, USA) and latency to the first paw flick (left/right, fore/hind) was recorded in seconds. For the tail-flick test, animals were gently restrained in a towel, and the mid-portion of its tail was placed beneath a radiant light source (Columbus Instruments). Heat was applied via focused light, and tail withdrawal latency was recorded. This sequence - hotplate followed by tail flick - was repeated at 0, 15, 30, 60, 90, 120, and 240 minutes.

### Statistical analysis

We statistically analyzed differences due to diet and variation in dietary obesity (that is, low gainer (LG) vs. high gainer (HG)) using a hierarchical analysis of variance. The first level compared control and high-fat diet groups. The second level compared how variation in dietary obesity (that is, LG vs. HG) affected osteoarthritis outcome measurements. In addition, we evaluated how the variation in dietary obesity affected osteoarthritis outcomes by conducting an analysis of covariance, using percentage body fat as the covariate. To assess the relative effect of diet, percentage body fat, and knee osteoarthritis on biomechanical, neurobehavioral, and inflammatory outcomes, we constructed bivariate and multivariate generalized linear models to identify which variables (that is, diet, percentage body fat, or knee osteoarthritis) remained independently associated with the outcome measures in the multivariate model. Statistical significance was reported at the 95% confidence level (*P *< 0.05), and the multivariate analyses repeated-testing error was controlled for using a 5% false discovery rate correction [35]. Statistical analyses were conducted using JMP 8.0 (SAS Institute, Cary, NC, USA).

## Results

### Variation in susceptibility to diet-induced obesity determines progression of osteoarthritis

**Figure 1:**
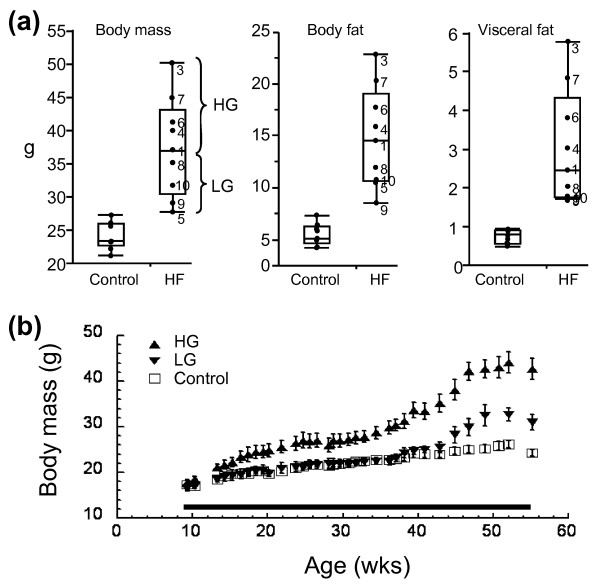
**Diet-induced changes in body mass and fat levels in control and high-fat fed mice**. **(a) **High-fat (HF)-fed mice showed much greater levels of variation in body mass, body fat, and visceral fat compared with control mice. The same individual HF mice (denoted numerically) fell in either the upper half or lower half of the bar plot distributions for these variables. Those mice in the upper half of the distribution were classified as high gainers (HG), and those in the lower half were classified as low gainers (LG). **(b) **Body mass in HG mice was greater than controls after 4 weeks of HF feeding compared with 37 weeks of HF feeding in LG mice (*P *< 0.05). Bar indicates duration of HF feeding. Data shown as mean ± standard error of the mean.

C57BL/6 mice are prone to dietary obesity and the metabolic disorders associated with obesity; however, recent studies have documented a significant amount of phenotypic variation in the response of C57BL/6 to high-fat feeding [28,36]. To characterize susceptibility to diet-induced obesity, we examined the body mass, body mass gain, body fat, and visceral fat following 45 weeks of feeding mice either a control chow or a high-fat chow diet (Figure 1a). All high-fat-fed mice had greater body mass, body mass gain, body fat, and visceral fat than the control-chow-fed mice, indicating that all mice fed the high-fat diet were susceptible to diet-induced changes in adiposity. The coefficient of variation for each of these four indices, however, was approximately double for the high-fat-fed mice compared with that for the control-fat-fed mice. High-fat feeding thus amplified the normal variation in body mass and fat.

Within the high-fat-fed mice, specific individual mice fell in the top half of the distribution for body mass, body mass gain, body fat mass, and visceral fat mass (Figure 1a); these mice were thus labeled HG mice. Mice that fell in the bottom half of the distribution were labeled LG mice. When body mass was compared between the HG mice and LG mice over time, the HG mice had significantly greater body mass than controls after about 4 weeks of high-fat feeding, whereas the LG mice did not develop significantly greater body masses than controls until after about 38 weeks of high-fat feeding (Figure 1b). Body mass was thus elevated relative to controls for 41 weeks in HG mice and for 7 weeks in LG mice - a nearly sixfold greater cumulative time course of elevated body mass in HG mice versus LG mice.

**Figure 2:**
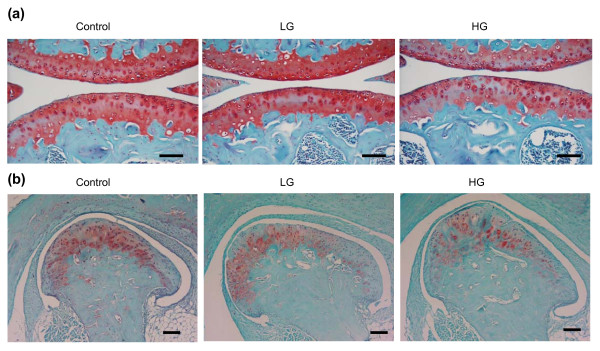
**Increased osteoarthritic changes in high-fat-fed high gainer mice**. **(a) **Representative histological images of knee joints showing increased proteoglycan depletion in high gainer (HG) mice as indicated by a loss of the red safranin-O staining. Scale bar = 100 μm. **(b) **Representative histological images of temporomandibular joints in control, low gainer (LG) and HG mice. There is a nonsignificant trend (*P *= 0.10) for increased loss of safranin-O staining in LG mice and HG mice. Scale bar = 100 μm.

We focused on the incidence of knee osteoarthritis with dietary obesity since the knee joint is the primary joint affected by obesity in humans and significant spontaneous osteoarthritis of the knee occurs in mice. HG mice showed a significant increase in the incidence of knee osteoarthritis due to a loss of cartilage matrix proteoglycans as indicated by a loss of safranin-O staining (Figure 2a and Table 1). Susceptibility to diet-induced obesity directly affected safranin-O staining intensity, with LG mice being protected from loss and HG mice having accelerated loss compared with controls (Table 1). In fact, among mice fed a high-fat diet, 90% of the variation in loss of cartilage proteoglycan staining intensity was explained by variation in body fat (*r*^2 ^= 0.90, *P *< 0.01).

**Table 1:** Knee joint histology, tibial cartilage material property, and trabecular bone osteoarthritis outcomes

Parameter	Control	LG	HG	Diet (*P *value)	Diet × percentage body fat (*P *value)
Knee modified Mankin score	18.2 ± 1.5	15.8 ± 2.5	25.1 ± 1.5*	0.17	**0.003**
Cartilage degeneration	4.4 ± 0.7	5.1 ± 0.9	6.7 ± 0.9	0.11	0.47
Safranin-O loss	7.7 ± 1.0	3.8 ± 0.3*	11.7 ± 0.8*^#^	0.66	**<0.001**
Tidemark duplication	0.22 ± 0.11	0.16 ± 0.10	0.13 ± 0.13	0.60	0.29
Chondrocyte cloning	0.67 ± 0.22	0.42 ± 0.16	1.1 ± 0.3	0.70	0.14
Hypertrophic chondrocytes	1.8 ± 0.3	1.3 ± 0.7	2.1 ± 0.4	0.88	0.18
Fibrocartilage	0.04 ± 0.04	0.33 ± 0.33	0 ± 0	0.48	0.34
Relative subchondral bone thickness	3.4 ± 0.3	4.7 ± 0.5	3.5 ± 0.7	0.26	0.31
Aggregate modulus (H_A_)	1.49 ± 0.18	2.18 ± 0.03	1.74 ± 0.20	**0.003**	0.10
Permeability (× 10^-16^, m^4^/N-s)	2.38 ± 0.67	1.90 ± 0.32	1.80 ± 0.60	0.48	0.77
Relative tibial epiphysis trabecular bone volume	0.43 ± 0.02	0.51 ± 0.07	0.40 ± 0.03	0.56	0.72

Statistical differences among control, low gainer (LG), and high gainer (HG) values were determined with a hierarchical analysis of variance. Overall diet and diet × percentage body fat effects were analyzed by analysis of covariance. *P < 0.05 compared with control values. #*P *< 0.05 for HG versus LG values. *P *values less than 0.05 shown in bold.

The onset of osteoarthritis, due in part to the loss of proteoglycan content in cartilage, is characterized by changes in the material properties of the articular cartilage. These changes typically include a decrease in the tissue aggregate modulus and an increase in fluid permeability [37], which we measured on the medial tibial plateau using a micro-indentation test [30]. The aggregate modulus was significantly increased in mice fed a high-fat diet (Table 1), due in large part to the elevated modulus and proteoglycan content of the medial tibial cartilage matrix of the LG mice compared with controls. Moreover, consistent with the decreased proteoglycan content in the knee cartilage in HG mice versus LG mice, aggregate modulus decreased with increasing body fat in high-fat fed mice (*r*^2 ^= 0.64, *P *< 0.05). Neither diet nor susceptibility to dietary obesity significantly altered fluid permeability (Table 1). These observations indicate that a high-fat diet alters the material properties of articular cartilage by increasing the aggregate modulus in a mechanism closely tied to proteoglycan density. Furthermore, the onset of degenerative changes in HG mice, most notably proteoglycan loss, appears to at least partly revert the aggregate modulus to control levels.

We also examined the temporomandibular joint to determine whether a systemic factor, such as adipose-associated inflammation, contributes to the increased incidence of osteoarthritis at nonweight-bearing sites. There were no significant differences in cartilage structure with diet or between HG mice and LG mice (Figure 2b and Table 2). Although high-fat feeding did not significantly increase the loss of safranin-O staining intensity, a trend for this effect was observed (Table 2).

**Table 2:** Temporomandibular joint osteoarthritis scoring

Parameter	Control	LG	HG	Diet (*P *value)	Diet × percentage body fat (*P *value)
Temporomandibular Composite score	7.0 ± 0.4	8.6 ± 0.5	8.4 ± 1.7	0.16	0.77
Cartilage degeneration	2.7 ± 0.3	3.4 ± 0.3	2.9 ± 1.1	0.56	0.96
Safranin-O loss	4.2 ± 0.3	5.2 ± 0.6	5.5 ± 0.9	0.10	0.67

Comparisons with control values or between low gainer (LG) and high gainer (HG) high-fat diet-fed groups were not statistically significant (*P *> 0.05).

### Diet and adiposity alter functional biomechanical parameters independent of osteoarthritis severity

**Figure 3:**
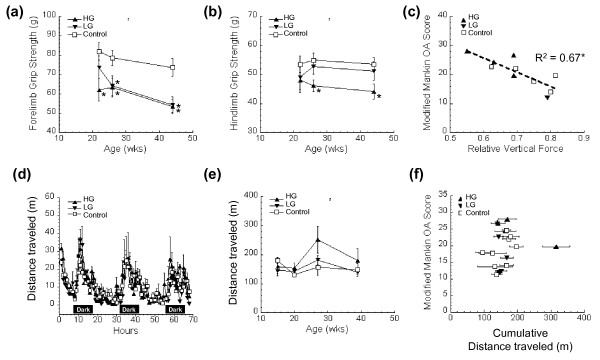
**Musculoskeletal performance in high-fat-fed mice**. **(a) **Fore-limb grip strength reductions in high-fat (HF)-fed mice over time (three measurements/animal/timepoint). **(b) **Hind limb grip strength reductions in HF-fed high gainer (HG) mice over time (three measurements/animal/timepoint). **(c) **Knee joint osteoarthritis (OA) scores were negatively correlated with the peak vertical component of the ground reaction force (expressed per unit body mass) from the hind limb during self-selected steady-speed locomotion. **(d) **Spontaneous horizontal distance traveled during a 72-hour period in control and HF-fed mice at 39 weeks of age. **(e) **Average horizontal distance traveled during a 10-hour dark period by control and HF-fed mice at different ages. **(f) **Comparison of knee OA score with the cumulative dark phase distance traveled (average of 15, 20, 27, and 39 weeks of age). Data shown as mean ± standard error of the mean. *P < 0.05 versus age-matched controls.

Osteoarthritis is associated with muscle weakness, impaired motor performance, and altered joint loading in human subjects. To assess how these factors are affected by a high-fat diet and correspond to osteoarthritis severity, we examined longitudinal changes in grip strength, locomotor coordination, gait, and spontaneous locomotor activity. Forelimb grip strength was significantly reduced in HG mice by approximately 25% compared with control mice after 13 weeks of high-fat feeding with no further changes occurring beyond this timepoint (Figure 3a). Forelimb grip strength also decreased after 13 to 17 weeks of high-fat feeding in LG mice and remained significantly lower throughout 35 weeks of feeding. After 17 weeks of high-fat feeding, hind limb grip strength significantly decreased in HG mice but not in LG mice relative to controls and remained weakened after 35 weeks of high-fat feeding (Figure 3b). Surprisingly, the strong negative association between grip strength and a high-fat diet (or percentage body fat) did not correspond to a negative association between grip strength and knee osteoarthritis (Table 3). A multivariate model indicates diet remained a significant covariate with grip strength when also accounting for percentage body fat and knee osteoarthritis score (Table 3).

**Table 3:** Biomechanical, neurobehavioral, and inflammatory changes with diet-induced obesity

	Bivariate (*r*)			Multivariate (β, *r*^2^)			
		
Parameter	Diet	Percentage body fat	Knee OA	Diet (β)	Percentage body fat (β)	Knee OA (β)	Whole model (*r*^2^)
Biomechanical							
Dark phase locomotion (m)	0.25	0.26	0.14	-	-	-	0.05
Velocity (cm/s)	-0.42*	-0.40	0.01	1.02	-0.11	0.14	0.21
Prompted velocity (cm/s)	-0.26	-0.19	0.09	-	-	-	0.09
Stride frequency (Hz)	-0.34	-0.37	0.08	-	-	-	0.22
Prompted stride frequency (Hz)	-0.31	-0.29	0.03	-	-	-	0.11
Rotarod latency to fall (s)	-0.71***	-0.83***	-0.33	-18.7	-10.1**	0.55	0.70***^a^
Forelimb grip strength (g)	-0.70***	-0.54*	-0.16	15.6**	0.73	-0.15	0.50**^a^
Hind limb grip strength (g)	-0.45*	-0.46*	-0.26	-	-	-	0.21
Neurobehavioral							
Hotplate withdrawal latency (s)	-0.63**	-0.55**	-0.31	2.24	0.076	-0.106	0.43*
Tail-flick withdrawal latency (s)	-0.02	-0.01	-0.13	-	-	-	0.02
Time in open areas (%total)^b^	-0.47*	-0.46**	0.01	2.56	-0.18	0.18	0.43*
Time freezing (%total)^b^	0.46*	0.32	0.33	-14.06*	-1.16	0.95	0.35*
Stretch attends^b^	-0.66***	-0.60**	-0.20	4.85	-0.03	-0.03	0.51**^a^
Open-closed transitions^b^	-0.18	-0.17	0.32	-	-	-	0.23
Time immobile^c ^(s)	-0.56**	-0.44	-0.30	63.1*	4.0	-3.34	0.37
Inflammatory							
IL-1α	-0.08	-0.09	-0.49*	18.8	3.0	-8.2*	0.27
IL-1 receptor antagonist	-0.05	-0.07	-0.18	-	-	-	0.03
IL-12	0.18	0.17	0.19	-	-	-	0.06
Keratinocyte-derived cytokine	0.17	0.17	-0.21	-	-	-	0.12
IFNγ-induced protein	0.10	-0.01	0.06	-	-	-	0.08
MIP-1α	0.24	0.39	0.17	-	-	-	0.22
Leptin	0.84***	0.95***	0.53**	-1.9	2.9***	1.1*	0.93***^a^
Adiponectin	-0.19	-0.02	-0.40	2694*	324*	-274**	0.42*

Inflammatory concentrations were measured in serum, and time points for comparisons are described in the text. MIP-1α, macrophage inflammatory protein-1; OA, osteoarthritis. ^a^*P *< 0.05 controlling for 5% false discovery rate correction of multivariate whole-model analyses. ^b^Zero-maze behavioral test. ^c^Tail suspension test. *P < 0.05, **P < 0.01, ***P < 0.001 using bivariate and multivariate generalized linear modeling. *P *values less than 0.05 shown in bold.

To further test the relationship between musculoskeletal force output and knee osteoarthritis, we conducted a kinetic gait analysis using a force plate to measure foot-ground reaction forces. The peak vertical force applied to the ground at mid-stance during trotting gaits approximates the maximal voluntary limb force during gait [38]. We found that the peak vertical force applied to the ground, normalized to body mass, was negatively related to the severity of osteoarthritic changes in the knee (Figure 3c). This finding suggests that modulating limb force is functionally related to the severity of knee osteoarthritis. One behavioral change that mice may use to reduce ground reaction forces is decreasing gait velocity. In fact, self-selected gait velocity was slower in 35-week-old mice fed a high-fat diet (Table 3). This reduction was not correlated with percentage body fat or knee osteoarthritis (Table 3), and it did not occur in conjunction with other gait changes, such as stride frequency or prompted gait conditions.

A potential confounding factor in examining the relationship between obesity and osteoarthritis is the effect of either obesity or osteoarthritis on spontaneous activity levels. Joint unloading lowers cartilage proteoglycan content and structure, whereas remobilization of joints and exercise-stimulated joint loading increases cartilage thickness and proteoglycan content [39-41]. Spontaneous locomotion, indicated here as horizontal distance traveled, was not significantly different among the control mice, LG mice, or HG mice over a 72-hour period at four different time points of high-fat feeding (Figure 3d,e). Susceptibility to diet-induced obesity does not therefore appear to be mediated by differences in the levels of spontaneous locomotion. When averaged across all time points, nearly all of the mice showed the same level of spontaneous activity despite a more than twofold variation in knee osteoarthritis severity (Figure 3h).

The observation that HG mice have reduced strength but normal spontaneous activity levels suggests that conditions which challenge the musculoskeletal system beyond normal activities may reveal impaired motor function. In the clinical setting, functional impairment is also assessed with physical activity challenges, such as a sit-to-stand test or a 6-minute walk test. For mice, we measured the latency to fall using a rotarod test to determine whether diet-induced obesity impaired motor function. There were no significant differences in latency to fall after 21 weeks of high-fat feeding (*P *= 0.79); however, after 34 weeks of high-fat feeding, performance decreased with a high-fat diet and in proportion to percentage body fat (Table 3). A multivariate analysis indicates that percentage body fat remains a significant predictor of impaired performance, when accounting for diet and knee osteoarthritis score (Table 3). The time course of this decrease in motor performance indicates that decreased motor performance occurs subsequent to the decrease in grip strength, suggesting that muscle weakness precedes impaired musculoskeletal function associated with diet-induced obesity in mice.

### Pain-sensing and coping behavioral impairments due to high-fat feeding

**Figure 4:**
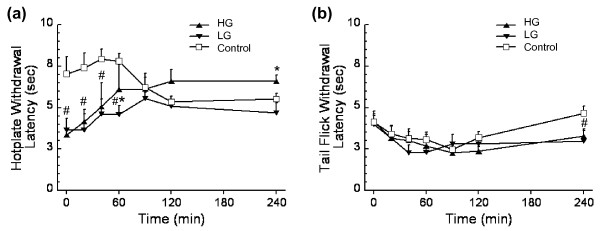
**Central thermal hyperalgesia in high-fat-fed mice at 53 weeks of age**. **(a) **Paw-withdrawal latency durations versus time for a hotplate test of centrally-mediated thermal hyperalgesia. High-fat (HF)-fed mice showed thermal hyperalgesia for the first 60 minutes of hotplate testing. **(b) **Tail-flick latency over time for a test of peripherally-mediated thermal hyperalgesia. HF-fed mice showed thermal hyperalgesia at 240 minutes of tail-flick testing. Data shown as mean ± standard error of the mean. *P < 0.05 for comparison with time-matched control value for either the low gainer (LG) group or the high gainer (HG) group. #*P *< 0.05 for time-matched HF diet versus control comparison.

Pain perception involves an interplay among nociceptive factors that mediate the intensity of the afferent signal and behavioral factors that excite or suppress this nociceptive response [21,22]. The effect of a high-fat diet on nociceptive behavioral responses was assessed via two acute thermal pain tests, the hotplate and tail-flick tests. These tests provided insight into nociceptive mechanisms generally believed to involve primarily supraspinal and spinal pathways, respectively [42]. The withdrawal latency for the hotplate test over the first 60 minutes of testing was significantly faster in LG mice and HG mice, compared with control mice (Figure 4a). With repeated testing, the withdrawal latency for the high-fat-fed mice became prolonged such that, by 100 minutes after the first test, the withdrawal latencies were not different from the controls. The bivariate associations between the initial withdrawal latency and diet or percentage body fat disappeared in the multivariate analysis that included diet, percentage body fat, and knee osteoarthritis score, indicating that neither diet or percentage body fat was independently related to the thermal hyperalgesia (Table 3). For the tail-flick test, there were no differences in withdrawal latencies over the first 120 minutes of testing (Figure 4b). By the 240-minute time point, however, the withdrawal latencies of the high-fat-fed mice were significantly faster than controls.

Affective behavioral traits, such as anxiety-like and depressive-like behaviors, may result from chronic pain or may contribute to an impaired ability to cope with exposure to painful stimuli. Anxiety-like responses were assessed in the zero maze after 41 weeks of feeding in control mice, LG mice, and HG mice that were naïve to the maze [31]. High-fat-fed animals spent less time in the open areas of the maze and more time in freezing postures (Table 3). High-fat-fed animals also displayed fewer stretch attend postures, although there was no significant difference in open-closed area transitions (Table 3). The bivariate associations between diet and freezing behavior remained in the multivariate analysis, indicating that diet was independently related to this behavior even when controlling for percentage body fat and knee osteoarthritis score (Table 3). Behaviors were also assessed with tail suspension, where increased immobility time indicates a reduction in antidepressive-like behavior [43]. High-fat fed animals were significantly less immobile during the test (Table 3). Furthermore, there was no significant association with percentage body fat in the bivariate and multivariate models, indicating that a high-fat diet - rather than the degree of dietary obesity - mediates their antidepressive-like behaviors.

### Systemic adipokines, diet-induced obesity, and osteoarthritis

Diet-induced obesity is associated with a shift in activities of proinflammatory and anti-inflammatory mediators that generally favor elevated tissue and systemic proinflammatory immune responses. Additionally, a number of proinflammatory cytokines have been implicated in the pathogenesis of osteoarthritis, including IL-6, IL-17, and TNFα. Serum levels of these cytokines were below the lowest level of quantification for many animals in a manner that was independent of the diet group, and thus were not reported. Serum concentrations of other detectable cytokines and chemokines, such as IL-12, keratinocyte-derived cytokine, IFNγ-induced protein, and macrophage inflammatory protein-1α, were not independently associated with changes in diet, percentage body fat, or knee osteoarthritis score (Table 3). IL-1α is a critical proinflammatory cytokine involved in cartilage catabolism and the pathogenesis of type 2 diabetes [44,45]. A high-fat diet and percentage body fat were not significantly associated with IL-1α levels (Table 3). The serum IL-1α concentration, however, was negatively associated with knee osteoarthritis score in both a bivariate and a multivariate analysis (Table 3). This finding was not associated with changes in IL-1-receptor antagonist levels, which were not altered by diet, percentage body fat, or knee osteoarthritis severity (Table 3).

**Figure 5:**
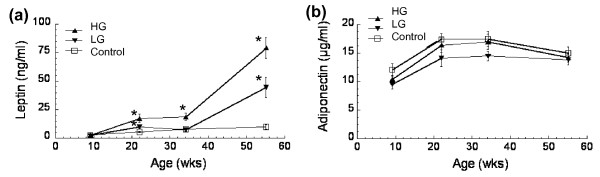
**Longitudinal serum adipokine concentrations in control and high-fat-fed mice**. **(a) **Leptin and **(b) **adiponectin concentrations at 9, 22, 34, and 54 weeks of age. Data shown as mean ± standard error of the mean. *P < 0.05 versus age-matched controls. HG, high gainer; LG, low gainer.

Leptin, an adipokine with proinflammatory activity, was increased systemically in high-fat-fed mice following a pattern that was similar to the temporal changes in body mass (Figure 5a). Adipose tissue is the primary source of leptin production, and at the final time point the serum leptin concentrations per unit fat mass were 1.75 ± 0.30 ng/ml/g, 4.18 ± 0.67 ng/ml/g, and 4.39 ± 0.44 ng/ml/g fat for control mice, LG mice, and HG mice, respectively. The higher fat-mass-specific leptin concentrations in high-fat-fed mice are consistent with the development of leptin resistance in both LG mice and HG mice [46]. At the final time point, leptin concentrations were independently associated with a high-fat diet, body fat, and knee osteoarthritis levels (Table 3). After controlling for interactions among these variables with a multivariate model, leptin remained significantly associated with percentage body fat and the knee osteoarthritis score (Table 3).

The anti-inflammatory adipokine, adiponectin, is typically reduced with adipocyte hypertrophy and increased adiposity. Serum adiponectin concentrations were not different between high-fat-fed mice and control-fed mice, although there was a trend for reduced values with high-fat feeding (Figure 5b). Adiponectin levels peaked between approximately 20 and 35 weeks of age, and by 54 weeks of age the levels were very similar between control mice and high-fat-fed mice (control = 14.9 ± 1.1 μg/ml; high fat = 14.0 ± 0.52 μg/ml). Per unit fat mass, however, serum adiponectin concentrations at the final time point were significantly reduced with high-fat feeding: 2.79 ± 0.20 μg/ml/g fat, 1.34 ± 0.11 μg/ml/g fat, and 0.79 ± 0.04 μg/ml/g fat in the control mice, LG mice, and HG mice, respectively. Although adiponectin concentrations were not independently associated with diet, percentage body fat, or knee osteoarthritis, adiponectin was negatively associated with knee osteoarthritis scores when controlling for diet and body fat (Table 3).

### Effect of leptin, IL-1, and fatty acids on chondrocyte matrix homeostasis

**Figure 6:**
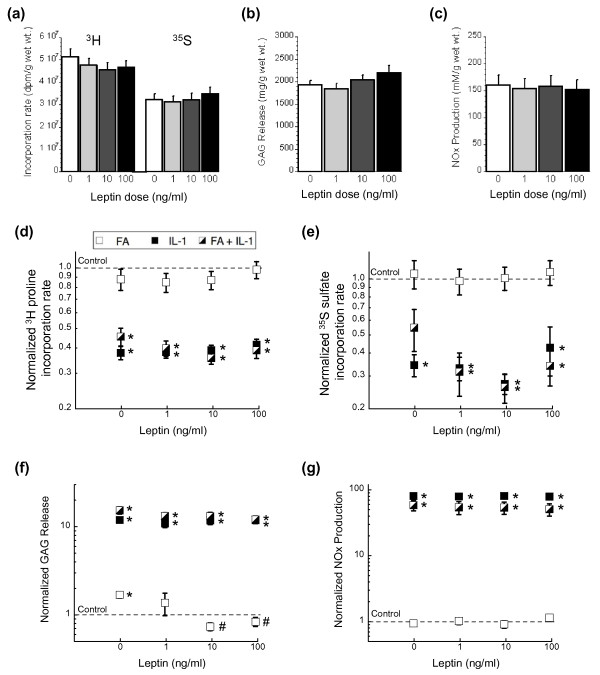
**Effect of physiologic leptin ± IL-1α and fatty acid on healthy cartilage extracellular matrix homeostasis**. Forty-eight-hour *in vitro *tissue culture experiments were performed on macroscopically intact porcine femoral articular cartilage explants to determine the acute effect of leptin stimulation on extracellular matrix synthesis and degradation. **(a) **Effect of leptin stimulation on collagen and sulfated glycosaminoglycan (S-GAG) synthesis rates determined by radioisotope incorporation of [^3^H]proline and [^35^S]sulfate, respectively (N = 9 joints, n = 5 explants per joint). Data are normalized to the average control value per joint. **(b) **S-GAG release from cartilage explants due to leptin stimulation (N = 12, n = 5). **(c) **Nitrite and nitrate (NOx) production from cartilage explants due to leptin stimulation (N = 9, n = 5). Leptin-stimulated conditions were not significantly different from control values (*P *> 0.05). Effect of leptin stimulation on cartilage **(d) **collagen synthesis, **(e) **S-GAG synthesis, **(f) **S-GAG release, and **(g) **NOx production when co-treated with 0.5 mM fatty acids (FA) or 0.1 ng/ml IL-1α separately or combined (N = 3, n = 5). FA treatment was a 1:1 ratio of palmitic:oleic acids. *y *axes are on a log scale. *P < 0.05 versus untreated control (dashed line). #*P *< 0.05 versus zero leptin condition. Data shown are mean ± standard error of the mean.

The significant relationship between adiposity, leptin, and osteoarthritis in high-fat-fed mice is consistent with previous reports of the proinflammatory actions of leptin in cartilage [16-18,47-49]. To test the potential role of leptin in the pathogenesis of osteoarthritis, an *in vitro *study was conducted using porcine cartilage explants to determine the effects of physiologic levels of leptin on cartilage matrix turnover and inflammation. Leptin did not significantly affect the incorporation of [^3^H]proline or [^35^S]sulfate (Figure 6a), indicating that leptin did not alter collagen or S-GAG synthesis (major components of the extracellular matrix). Furthermore, neither S-GAG release nor NO_x _production were altered by leptin (Figure 6,c).

Given the lack of an independent effect of leptin on extracellular matrix synthesis, degradation, or NO_x _production, we examined whether leptin altered extracellular matrix synthesis or degradation when cartilage explants were co-stimulated with IL-1α (1 ng/ml) with or without a high concentration of fatty acids (0.5 mM 1:1 palmitic:oleic fatty acids). The [^3^H]proline (Figure 6d) and [^35^S]sulfate (Figure 6e) incorporation rates were reduced by more than 50% by IL-1α treatment compared with unstimulated controls. Fatty acid treatment alone did not affect incorporation rates, and the combined IL-1α plus fatty acid treatment had no effect on [^3^H]proline incorporation rates (Figure 6d) and a slight positive effect on [^35^S]sulfate incorporation rates compared with IL-1α treatment alone (Figure 6e). The addition of leptin had no affect on [^3^H]proline incorporation rates, indicating that leptin does not synergistically mediate IL-1α-stimulated reductions in collagen synthesis. Leptin had no affect on IL-1α-mediated reductions in S-GAG synthesis, as indicated by the [^35^S]sulfate incorporation rates (Figure 6e). Leptin did, however, reduce [^35^S]sulfate incorporation rates in IL-1α and fatty acid co-stimulated explants from approximately 50% to 30% of the control rate (*P *< 0.05), negating a slight protective effect of fatty acids on IL-1α-mediated reductions in S-GAG synthesis.

Leptin also mitigated the fatty-acid-stimulated S-GAG release from the explants (Figure 6f). Fatty acid treatment increased S-GAG release by about 70% relative to control, and the two highest leptin treatments (10 and 100 ng/ml) returned the S-GAG release to control levels (Figure 6f). Similarly, leptin mitigated S-GAG release in IL-1α and fatty acid co-stimulated explants, from a more than 15-fold increase in the absence of leptin down to a 12-fold increase with the highest leptin dose. IL-1α-mediated S-GAG release, which was increased nearly 12-fold, was unaffected by leptin treatment.

NO_x _production was increased approximately 80-fold with IL-1α treatment (Figure 6g) and, unlike previous reports showing a synergistic effect of leptin on IL-1α-mediated NO_x _production [48], we did not observe an effect of leptin on IL-1α-mediated NO_x _production using a physiologic range of leptin doses. IL-1α and fatty acid co-stimulation treatments generated NO_x _levels that were lower than IL-1α treatment alone (Figure 5g), and fatty acid treatment by itself had no affect on NO_x _production. Furthermore, treatment with leptin did not alter NO_x _production in explants treated with fatty acids. Leptin mitigation of changes in fatty acid-stimulated S-GAG release thus does not appear to involve nitric-oxide-dependent pathways.

## Discussion

Obesity is a risk factor for osteoarthritis in joints throughout the body, especially the knee. The relative contribution of biomechanical, behavioral, and inflammatory factors to the symptomatic and structural pathogenesis of obesity-associated osteoarthritis, however, is not well understood [50]. In the present article, we show that a high-fat diet induces osteoarthritic changes in the knee joint in proportion to fat gain following 45 weeks of high-fat feeding in female C57BL/6J mice. Fat gain, which varied considerably in response to a high-fat diet as previously reported [28], strongly correlated with cartilage proteoglycan loss and reduced the aggregate modulus in high-fat-fed mice. Resistance to diet-induced obesity protected mice from the normal age-associated loss of cartilage proteoglycan content, whereas susceptibility to diet-induced obesity significantly increased the loss of cartilage proteoglycan content. Differences in body weight alone do not explain this observation, as LG mice weighed more than the control-diet mice yet they had reduced proteoglycan loss. Susceptibility to diet-induced obesity has been attributed to epigenetic changes in adipose tissue gene expression, including genes involved in the Wnt signaling pathway [28]. Furthermore, differences in social status (for example, dominant vs. submissive) under chronic stress conditions may also affect weight gain in response to high-fat feeding [51]. It is not known whether these conditions are mechanistically related to the observed changes in knee osteoarthritis.

The findings that the fat content of the diet mediates the relationship between body weight, adiposity, and knee osteoarthritis in C57BL/6J mice suggest that obesity-related risk factors for osteoarthritis are sensitive to environmental factors that regulate metabolism. While there is some evidence supporting a direct link between the regulation of lipid metabolism and osteoarthritis [52-54], increased adiposity may promote the development of osteoarthritis indirectly through changes in biomechanical and/or inflammatory pathways.

In the current study, musculoskeletal strength, locomotor speed, and motor coordination, but not spontaneous locomotor activity, were significantly impaired by high-fat feeding. These effects were shown with respect to reduced grip strength, slower self-selected speeds, and the significant reduction in rotarod-tested motor coordination and function. A similar relationship occurs in humans, in which quadriceps muscle weakness is a risk factor for incident, but not progressive, knee osteoarthritis in older obese women [5,55]. Quadriceps muscle weakness increases the risk of incident knee pain [56] and is associated with increased pain and reduced function in individuals with knee osteoarthritis [57]. Reduced physical function and the use of slower walking speeds in humans with osteoarthritis are generally attributed to reduced joint stability, altered neuromuscular function, and increased pain [58-61]. In the current study, reduced grip strength occurred at a much earlier time point compared with changes in motor coordination and gait, suggesting that neuromuscular deficits associated with muscular force generation are an initiating factor in the pathogenesis of obesity-associated osteoarthritis.

The lack of association between spontaneous locomotor activity and the development of osteoarthritis in control mice, LG mice, and HG mice is notable for several reasons. First, differences in spontaneous activity do not correspond to differences in the degree of weight and adipose tissue gain (that is, LG vs. HG). These data suggest that differences in susceptibility to weight gain in HG mice relative to LG mice are not explained by reduced energy expenditure in HG mice, which is consistent with a previous finding that differences in resting metabolic rate are compensated by differences in food intake in high-fat fed C57BL/6 mice [36]. Second, no association occurred between the average horizontal distance traveled and the incidence of knee osteoarthritis among control mice, LG mice, and HG mice. Similarly in humans, the habitual recreational activity level neither increases nor decreases the risk of developing symptomatic osteoarthritis in normal and overweight individuals [62].

An important factor in the interpretation of the behavioral results from this study is the well-recognized complexity - and the lack of predictability - of the relationships between structural changes and pain in osteoarthritis clinically [63]. In fact, previous clinical studies have shown that osteoarthritic disability is most highly associated with pain, obesity, and anxiety, with little relationship to structural changes (as measured radiographically) [64]. Neurobehavioral factors associated with osteoarthritis pain, including anxiety-like responses and nociception, were altered with a high-fat diet and increasing adiposity. In rats, acute exposure to a high-fat diet is perceived as a stressor, comparable with significant chronic or acute stress [65]. Stress is a common trigger for mood disorders [66], and a recent large-scale study has shown a clear relationship between obesity, obesity-related co-morbidities, and the prevalence of current or lifetime depression and anxiety, particularly in women [67].

Although there are many unknowns about the relationship between obesity and mood, such as the direction of causality and the interaction of environmental and genetic factors, mood disorders have a significant impact on disease severity (for example, pain and disability) in individuals with osteoarthritis. In women, both anxiety and depression predict weekly changes in osteoarthritis pain, with the effect of anxiety being twice as large as depression [68]. In mice, we observed a similar relationship between anxiety-like behavior and hyperalgesia. Female LG and HG mice showed increased thermal sensitivity for the hotplate test, but not the tail-flick test, suggesting that supraspinal sites are the primary neurological targets of modification by high-fat diets. Indeed, two recent studies have highlighted the role of leptin and urocortin 2 signaling in the brain as sites of action that modulate thermal nociception [69] and anxiety-like behavior [70], respectively. The current findings indicate that a high-fat diet may be an important environmental stimulus for modulating neurobehavioral factors associated with osteoarthritis pain, such as anxiety and hyperalgesia.

The findings of the present study also indicate that systemic concentrations of various proinflammatory cytokines are not significantly altered by a high-fat diet. Indeed, leptin was the only cytokine or adipokine that was altered by the high-fat diet. These findings are consistent with previous studies of high-fat diet-induced obesity in mice, which show significant inflammatory responses within the adipose tissue but generally mild systemic increases in serum cytokine concentrations [71]. For example, C57BL/6N mice fed a 35% fat diet for 10 weeks showed no changes in serum IL-1α, IL-1β, IL-4, IL-10, or TNFα, but had a significant, although low (7%), increase in IL-6 levels relative to controls [72].

Despite minimal effects of a high-fat diet on proinflammatory cytokine levels, statistically controlling for the effect of diet and percentage body fat revealed that systemic concentrations of leptin, adiponectin, and IL-1α are predictive of knee osteoarthritis severity. The mechanisms relating reduced IL-1α and adiponectin levels with joint degeneration remain unclear as osteoarthritis severity and extracellular matrix degradation is associated with increased levels of both IL-1α [73] and adiponectin [74]. For leptin, several previous studies provide support for an indirect role of leptin in osteoarthritis pathogenesis [75]. For example, recent studies have shown that mice deficient in leptin or the leptin receptor undergo extreme weight gain but exhibit no changes in osteoarthritis [76]. Furthermore, several *in vitro *studies have suggested that high levels of leptin can induce IL-1 expression or can act synergistically with IL-1 to induce NOS2 expression [17,48].

To directly examine whether increased levels of leptin can induce degenerative or proinflammatory changes in healthy cartilage, we treated cartilage explants with a range of physiologic doses of leptin, in the presence or absence of IL-1α and palmitic and oleic fatty acids. We found little or no effect of leptin on matrix biosynthesis, proteoglycan breakdown, or nitric oxide production *in vitro *(Figure 6), whereas the proinflammatory effects of leptin are apparent at superphysiologic concentrations [48,49]. Osteoarthritis increases the expression of leptin and leptin receptors in chondrocytes [49], suggesting that physiologic levels of leptin may mediate the production of inflammatory mediators in osteoarthritic but not normal tissue. There is evidence that the regulation of leptin expression through environmental, genetic, or epigenetic factors may be responsible for influencing the production of proinflammatory mediators and matrix metalloproteinases in cartilage [47]. Alternatively, leptin may indirectly alter chondrocyte activity through altered bone remodeling [77] or lipid metabolism [78].

The findings of the present study suggest several potential treatment targets for osteoarthritis in obese patients. As the high-fat diet itself, independent of a gain in body fat, was associated with several factors associated clinically with osteoarthritis (that is, altered cartilage properties, grip strength, increased anxiety-like behavior, and adiponectin level), our findings suggest that diet modification, in and of itself, could potentially reduce some of the known risk factors for osteoarthritis-associated disability. While there are few clinical data currently available to support this notion directly, previous studies have shown beneficial and anti-inflammatory effects of a diet low in arachidonic acid in patients with rheumatoid arthritis [79,80]. Strengthening exercises for the quadriceps to counter muscle weakness associated with obesity may also have therapeutic benefits for osteoarthritis pain [81]. In particular, recent studies have shown that knee strengthening exercises may be particularly beneficial for overweight and obese patients with osteoarthritis [82]. Ultimately, the greatest benefit with respect to pain and disability for obese osteoarthritis patients may come from a combination of knee strengthening coupled with moderate exercise and weight loss [83].

The significant associations between osteoarthritis severity and serum leptin, adiponectin, and IL-1α concentrations, independent of diet and adiposity, support the role of systemic adipokines as mediators of obesity-associated osteoarthritis. The strong association between leptin and disease severity, coupled with the recent observation that obesity due to the impairment of leptin signaling does not cause osteoarthritis in mice [76], suggests that leptin itself may be a target for osteoarthritis in obese patients. Given the pleiotropic effects of leptin in regulating appetite, skeletal metabolism, fertility, and many other physiologic functions, however, targeting leptin directly may prove overly complex as an osteoarthritis therapy. On the other hand, increased leptin levels are associated with increased concentrations of other proinflammatory cytokines such as IL-1, IL-6, IL-8, TNFα, and prostaglandin E_2 _[84], which may provide more specific and selective approaches for pharmacologic intervention in obesity-induced osteoarthritis.

While the use of a diet-induced obese mouse model provides numerous advantages for studying obesity and osteoarthritis - such as allowing for repeated testing and controlling for environmental conditions, diet, and age - there are limitations of its use for translational relevance. For example, it was not possible to examine biomechanical factors, such as limb alignment and net adduction moment about the knee, or other neuromuscular measurements involving proprioception and maximal knee extensor strength tests, which are associated with knee osteoarthritis in humans. Furthermore, the methods used to assess hyperalgesia and pain-related behaviors are not specific to the joints and do not include pressure-based stimuli. In general, the use of mouse models imposes limitations associated with animal size as well as challenges for translating clinical functional and behavioral tests that require knowledge about a particular cognitive state (for example, motivation or emotion). Nonetheless, our findings of osteoarthritis-like changes in this model provide further support for use of the mouse to study various molecular, biomechanical, and behavioral factors in the pathogenesis of joint degeneration using genetically-modified or inbred mice [85-87], diet-induced obesity [27,88-90], or joint injury [91].

## Conclusions

Our findings show that a high-fat diet induces a unique suite of biomechanical, neurobehavioral, and inflammatory changes associated with structural and symptomatic osteoarthritis in mice. Of these changes, systemic leptin, adiponectin, and IL-1α levels remain significantly associated with knee osteoarthritis severity when statistically controlling for the effects of diet and adiposity. Physiologic levels of leptin do not alter extracellular matrix homeostasis in healthy cartilage, suggesting that leptin may be a secondary mediator of cartilage degeneration in osteoarthritis. These findings provide new insights into potential pharmacologic, psychosocial, and physical therapies for the treatment or prevention of obesity-associated osteoarthritis.

## Abbreviations

BSA: bovine serum albumin; FBS: fetal bovine serum; HG: high gainer; IFN: interferon; IL: interleukin; LG: low gainer; NO_x_: nitrate and nitrite; S-GAG: sulfated glycosaminoglycan; TNF: tumor necrosis factor.

## Competing interests

The authors declare that they have no competing interests.

## Authors' contributions

TMG conceived of the study, oversaw all data collection and analysis, and drafted the manuscript. BF participated in the design, collection and analysis of data for the tissue culture experiments. JLH participated in data collection and analysis of cytokine and adipokine measurements. VBK contributed to the experimental design, analysis, and interpretation of the data. RMR and WCW contributed to the design, collection, analysis, and interpretation of the neurobehavioral data. LC participated in the collection and analysis of the cartilage material property data. LAS contributed to the experimental design and analysis of the cartilage material property data, and participated in the interpretation of all study results. FG contributed to the study conception, experimental design, data analysis and interpretation, and manuscript preparation. All authors have read and approved the final manuscript.
